# Exosomes in Clinical Laboratory: From Biomarker Discovery to Diagnostic Implementation

**DOI:** 10.3390/medicina61111930

**Published:** 2025-10-28

**Authors:** Majdi A. Aljohani

**Affiliations:** Department of Medical Laboratory Technology, Faculty of Applied Medical Sciences, University of Tabuk, Tabuk 71491, Saudi Arabia; ma.aljuhani@ut.edu.sa

**Keywords:** exosomes, EVs, liquid biopsy, preanalytical variability, non-invasive biomarkers

## Abstract

Exosomes, which are extracellular vesicles measuring 30–150 nm, are becoming a promising new target from cellular debris classification to a recognized biomarker with the potential to transform diagnostics. They have a fundamental role in intercellular communication, with selective molecular cargo that can reflect the pathophysiological state of parent cells. Exosomes are particularly advantageous for non-invasive liquid biopsies, as they provide continuous monitoring of disease progression or response to treatment. We detail the most recent diagnostic proteins, nucleic acids, and lipids in the context of different diseases. Here, we show the potential of exosomes as non-invasive biomarkers across diverse diseases, which may transcend the sensitivity of conventional biomarkers. The potential of exosome-based liquid biopsies to transform clinical laboratory practice will be determined by their ability to overcome challenges. Limitations comprise preanalytical variability, absence of standardized protocols, and heterogeneity in exosome isolation, which limit their diagnostic potential. The implementation is limited by isolation and analytical processes; however, many advanced platforms may offer multiplexed detection, which is accelerating their implementation process in clinical laboratories. Finally, we provide an overview of the clinical applications and preclinical advancements of exosomes to provide a perspective on the significance of exosomes for their use in biomarker study, as well as therapeutic monitoring in different diseases. Future initiatives must emphasize coordinated validation, economical scalability, and incorporation into clinical workflows to fulfill the potential of exosomes as advanced diagnostics.

## 1. Introduction

Exosomes are part of EVs, originate from endosomes, and range from 30 to 150 nm in size [1]. They play an important role in many cellular processes due to their unique biogenesis, molecular complexity, and functional versatility [2]. Extracellular vesicles were first introduced in 1971 when scientists tried to characterize vesicular structures produced by Ochromonas danica [3]. The term “exosome” was first used in 1981 to describe vesicles shed from the cell surface, but it was not until 1983 that exosomes were first discovered [4,5]. Their turning point concerned the 2013 Nobel Prize in Physiology or Medicine, which was awarded for the discovery of vesicle trafficking mechanisms [6,7,8].

**Figure 1:**
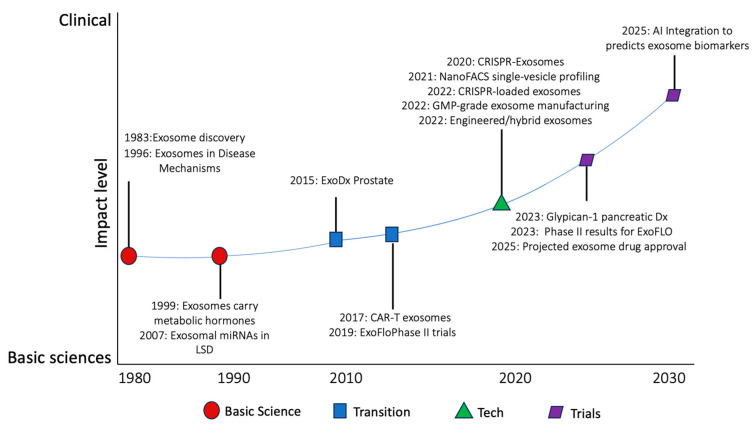
The major developments in exosome research and clinical development from 1980 to 2030, and the transition from basic science, illustrated by the horizontal axis, to clinical application, illustrated by the vertical axis.

This dramatic shift was driven by recent advances in analytical technologies. Critically, now we understand that the composition of exosomes is not solely a reflection of their cell of origin. Instead, it is actively influenced by selective cargo loading mechanisms that respond to the cell’s physiological or pathological state. This allows exosomes to function as precise carriers of molecular information [2,9], which makes exosomes dynamic signaling entities capable of reprogramming recipient cells at the genetic, metabolic, and functional level (Figure 1). Currently, exosomes are recognized not only as biomarkers but also as active participants in disease pathogenesis, diagnostics, and therapeutics. The transformation of exosomes from basic science to clinical application is summarized in Figure 1.

**Figure 2:**
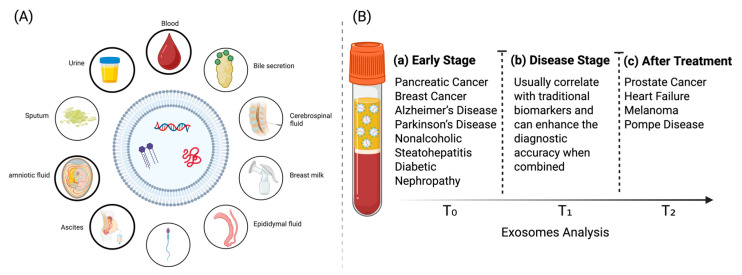
Multi-biofluid exosome biomarkers enable precision medicine across disease stages. (**A**) Exosomes are detectable in diverse biofluids, permitting non-invasive sampling. (**B**) Clinical utility spans (**a**) early-stage, (**b**) disease-stage, and (**c**) treatment monitoring.

Tissue and liquid biopsies have long been a vital aspect of disease diagnosis and are often used to characterize the disease and provide a treatment guide [10]. Nevertheless, traditional tissue biopsy techniques encounter many distinct barriers. These are a result of their invasiveness, cost, time, and sampling bias caused by tissue heterogeneity [11,12,13]. A liquid biopsy has a low abundance of biomarkers in plasma during the early phases of the disease in conventional biomarkers. Serum-derived exosomes are particularly advantageous for non-invasive liquid biopsies, as they provide continuous monitoring of disease progression or response to treatment (Figure 2B) [14,15,16]. Exosomes are detectable in more than 15 biofluids, including blood, urine, saliva, bile, cerebrospinal fluid, breast milk, epididymal fluid, semen, ascites, amniotic fluid, and sputum, making them ideal for non-invasive liquid biopsies (Figure 2A) [17,18,19]. Exosomes mirror the physiological and pathological state of their parental cells, as they have the ability to influence the transfer of proteins, lipids, and nucleic acids [20,21,22]. These exosomes are protected from enzymatic degradation by a lipid bilayer membrane, which guarantees the integrity of their content [23]. The sensitivity of exosome detection has been significantly enhanced in recent years. This is because of the application of high-sensitivity technologies to the science of exosome detection [24,25,26,27]. These significant characteristics make exosomes next-generation biomarkers with the potential to change diagnostics, disease monitoring, and early intervention in clinical practice.

Exosomes have become critical mediators of intercellular communication and disease biomarkers, thereby revolutionizing precision medicine and non-invasive diagnostics. Across numerous diseases, including cancer and neurological, cardiovascular, and metabolic disorders, exosomal biomarkers have demonstrated outstanding diagnostic and prognostic ability [19,22,28,29,30]. For example, exosomal PD-L1 has been predicted to predict immunotherapy response in melanoma and NSCLC [31,32,33]. Its predictive power is further enhanced when it is combined with CD28 in non-small cell lung cancer (NSCLC) patients [34]. Furthermore, exosomal glypican-1 shows beneficial early pancreatic cancer detection sensitivity [35]. In neurodegenerative diseases, exosomal tau and phosphorylated tau serve as biomarkers for Alzheimer’s and Parkinson’s disease, respectively [36,37,38]. Exosomal miR-1 has been linked in cardiovascular medicine to acute myocardial infarction [39,40] while exosomal HSP70 has been associated with heart failure and positively correlated to NT-proBNP (r = 0.493, *p* < 0.001) [41]. Supported by many clinical investigations, these results underline the increasing relevance of exosome-based diagnostics in precision medicine.

Beyond diagnostics, a lack of harmonized protocols hinders clinical translation due to technical challenges in exosome isolation, quantification, and standardization. These include variation in size, composition, and surface markers, which vary depending on their origins and physiological context [42,43]. Furthermore, the preanalytical variables play an important role in the variation. Moreover, the challenge for clinical laboratories is to balance the practical requirements of automated procedures, cost-effective scalability, and integration with existing analytical workflows with novel multi-omics approaches. However, recent advances in microfluidic platforms, size exclusion chromatography, and immunoaffinity capture enhance diagnostic potential with a low amount of sample with high accuracy [44,45]. These developments, however, do not overcome the fundamental standardization barriers preventing routine clinical implementation.

Despite these limitations, recently developed platforms are able to detect multiple molecular components simultaneously, including ExoLution Plus [46], ExoView [47], and biosensor platforms [48]. There is still a significant gap between the diagnostic potential and the routine use of exosomes in clinical laboratories. In compliance with the MISEV2023 recommendations, this review encompasses both exosomes and small extracellular vesicles (sEVs) in a broader sense, as both are pertinent to clinical laboratory diagnostic applications. This review investigates the biology of exosomes as diagnostic biomarkers in disease pathogenesis. It critically discusses their molecular composition and clinical applications across a variety of diseases, translational technologies for isolation and analysis, and the implementation challenges associated with clinical laboratories.

### 1.1. Exosome Biogenesis and Disruption in Disease

In the past few years, there has been significant progress in the understanding of the mechanisms of the formation and secretion of exosomes. Exosome biogenesis is a process that is highly regulated and, when disrupted, can lead to many disorders. Exosome biogenesis is initiated by early-sorting endosomes (ESEs) by endocytosis. After that, they mature into late endosomes and, subsequently, multivesicular bodies (MVBs). This serves as the initial sorting platform in the endocytic pathway [49,50]. Then, intraluminal vesicles (ILVs) develop within MVBs through two distinct mechanisms, which include ESCRT-independent and ESCRT-dependent pathways [51,52]. Initially, the endosomal sorting complexes necessary for transporting ESCRTs are recruited to the site of ILV formation. Exosome biogenesis begins with the inward budding of multivesicular body (MVB) membranes to form intraluminal vesicles (ILVs). These ILVs are released as exosomes upon MVB-plasma membrane fusion [2]. This process is tightly regulated through ESCRT-dependent mechanisms, where (ESCRT-0 to -III) complexes mediate ubiquitinated cargo sorting [53].

**Figure 3:**
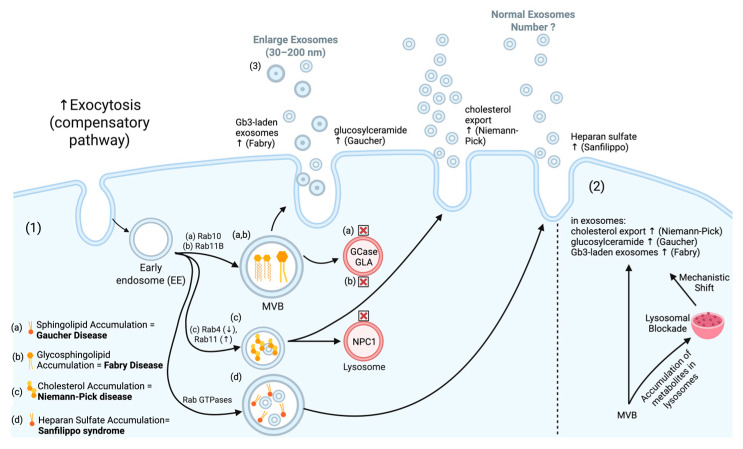
Schematic overview of exosome biogenesis and composition, highlighting the role of lysosomal diseases and the specific biomolecules that accumulate in exosomes in (a) Gaucher, (b) Fabry, (c) Niemann–Pick disease, and (d) Sanfilippo.

Several lysosomal storage diseases trigger a compensatory process in which the cell tries to evacuate accumulated substrates via exosomes. In Gaucher disease, glucosylceramide accumulation alters CD63/CD9 sorting. This accumulation leads to exosome hypersecretion and increased size heterogeneity [54,55,56]. Subsequently, during ILV formation, the endosome membrane undergoes a reorganization that results in a significant enrichment of selected proteins such as tetraspanins. This is an important process disrupted in Niemann–Pick Type C disease, where ceramide metabolism defects impair tetraspanin microdomain organization [57]. This prevents the sorting of cholesterol into ILVs for storage or exosomal release, resulting in in lysosomal cholesterol accumulation and compensatory exosome hypersecretion [58,59]. The same compensatory mechanisms occur in Fabry disease and Sanfilippo syndrome (Figure 3).

### 1.2. Clinical Laboratory Early-Stage Development

Despite two decades of comprehensive research evidencing the potential of exosomes, clinical implementation remains in early-stage development with limited FDA-approved application. Exosomes are currently acknowledged as critical mediators of intercellular communication. Consequently, they are transforming the clinical laboratory setting despite their practical limitations. They are the most promising candidates for non-invasive diagnostics for a variety of disorders. This potential is due to their ability to cross biological barriers, their minimal immunogenicity, their stability in body fluids, and their ability as detection biomarkers in tissue biopsies. Exosome-based liquid biopsies frequently exhibit superior sensitivity and specificity in early-stage identification when contrasted with conventional serum indicators, as evidenced by conditions like those of pancreatic cancer [60]. Although the CA19-9 biomarker is the gold standard for pancreatic cancer, it has many limitations, such as a reduced sensitivity in early-stage disease and difficulty in distinguishing pancreatic cancer from benign conditions [61,62,63]. These limitations are addressed by exosomal biomarkers, as cargo material is protected by a lipid biolayer, whereas free-circulating biomarkers may be susceptible to degradation. Moreover, they are capable of distinguishing between benign and cancerous conditions due to their disease-specific signatures, which include nucleic acids and proteins [35,64]. This early-stage development presents a challenge to traditional diagnostic methods. It demonstrates the significance of exosomes-mediated pathways, thereby generating new therapeutic targets and diagnostic opportunities.

This leap is evidenced by the ExoDx Prostate IntelliScore (EPI) test, which received an FDA breakthrough device designation for use in prostate patients. Recent studies demonstrate that ExoDx has a 91.3% negative predictive value (NPV) for high-grade prostate cancer, reducing unnecessary biopsies by ~30% [65,66]. Moreover, many clinical tests are under development, including the ExoTRU™ Urine Test, transplant rejection monitoring for kidney allograft rejection diagnosis, and others [67]. Despite the regulatory policies, this is not yet the universally accepted standard of care in the majority of clinical laboratories. Furthermore, it is not applicable for all diseases or all types of traditional biomarkers. FDA-preapproved and breakthrough-designated exosome tests are poised to be implanted in clinical laboratory [68]. This will offer the potential for earlier diagnosis, real-time therapy monitoring, and better patient outcomes. The potential of exosome-based liquid biopsies to transform clinical laboratory practice will be determined by their ability to overcome challenges. Exosome-based liquid biopsies may alter the standard of care across several medical fields as isolation and profiling technologies continue to progress.

## 2. Exosome Biology in Diagnostics

Exosomes are extracellular vesicles produced by all cells, carrying nucleic acids, proteins, lipids, and metabolites [9]. Exosomes can facilitate proximal and distal intercellular communication in health and disease, influencing many aspects of cell biology. Exosomes express a large range of antigen and receptor proteins, glycoproteins, and glycolipids on their surface, which initiate intracellular signaling pathways. Internally, exosomes package a variety of nucleic acids, such as mRNAs, miRNAs, rRNAs, lncRNAs, tRNAs, snRNAs, and snoRNAs [69,70,71]. Moreover, exosomes can also include double-stranded or single-stranded DNA and mitochondrial DNA [72]. Furthermore, their lumen contains signaling molecules such as cytokines, growth factors, small molecules, metabolites, and even functional enzymes [2]. Exosomes have the ability to transport proteins and genetic material between cells. This function has caused them to be increasingly leveraged in disease diagnostics as non-invasive biomarkers [73]. Many biomarkers in exosomes have been identified as promising tools for diagnosis. This includes mtDNA mutations in Parkinson’s disease and tau and phosphorylated tau (p-tau) in Alzheimer’s disease [37]. Public databases like ExoCarta [74], Vesiclepedia [75], EVpedia [76], and ExoBCD [77] have added numerous exosomal proteins, highlighting their molecular complexity. Moreover, ExMdb [78] contains exosomal nucleic acid biomarkers and disease–gene associations. Nevertheless, the realization of their diagnostic potential requires the harmonization of data standards and systematic validation of potential biomarkers.

### 2.1. Exosomal Membrane Proteins in Disease Pathogenesis

Exosome membranes contain specific surface molecules that when present in biofluids provide highly specific, non-invasive insights. Exosomes are significantly enriched in tetraspanins, a protein superfamily that organizes membrane microdomains known as tetraspanin-enriched microdomains (TEMs). This leads to the formation of clusters and interactions with a diverse array of transmembrane and cytosolic signaling proteins. Several tetraspanins, such as CD9, CD81, CD82, and CD63, are extensively employed as exosome markers and are involved in biogenesis, cargo selection, and cell targeting [2]. Furthermore, tetraspanin profiles can be distinguished from different cell types, offering tissue-specific signatures for diagnostic applications. For example, CD63 was found to be rich in melanoma exosomes [79], in contrast with CD9, which is rich in exosomes from oligodendrocytes [80]. Tetraspanins interact with integrins [81], ICAM-1, and major histocompatibility complex (MHC) class II proteins to facilitate the organization and sorting of proteins within exosomes [82,83]. Organotropic metastasis has been demonstrated to be determined by exosomal integrins. Emerging evidence demonstrates that in lung cancer patients, circulating exosomal integrin β3 can predict overall survival. Furthermore, it also functions as a quantitative predictor of the progression of brain-specific diseases [84]. Additionally, exosomal integrin can mediate the transfer of chemotherapy resistance between cancer cells [85].

The immunomodulatory potential of exosomal membranes is demonstrated by a complex array of checkpoint and regulatory proteins. Multiple studies have confirmed that exosomal PD-L1 contributes directly to immunosuppression while serving as a predictive biomarker for anti-PD-1 therapy response [85,86]. Another example is that of exosomal CD47, which mediates immune evasion in ovarian cancer [87]. Later research utilizing nanoflow cytometry to analyze overexpressed exosomal CD47 demonstrated significant immune evasion in vivo [88]. Moreover, based on many immune cell studies, exosomal MHC class II and I complexes can either promote or suppress immune responses [89,90]. Multiple cancer cell types have demonstrated the secretion of exosomes that contain CTLA-4 with retained immunosuppressive functionality [91]. Multiple studies have confirmed that exosomal TIM-3 is positively correlated with tumor progression, as it can be transferred between cells via exosomes [92]. An immuno-electron microscopy study confirmed that ICAM-1 mediates the adhesion requirements for exosome-induced T-cell suppression by exosomal PD-L [93,94,95]. Additional exosomal immune modulators include Galectin-9, which contributes to exosomes’ immunomodulatory functions [96]. Furthermore, checkpoint molecules such as exosomal CD40 [97] and TRAIL [98] modulate immune cell apoptosis and activation.

The diagnostic value of exosomal membrane proteins is extended to tissue-specific cancer biomarkers, including glypican-1 (GPC1), which provides a high sensitivity and specificity for pancreatic cancer [35,60]. Moreover, exosomal EpCAM can distinguish malignant from benign conditions, especially if it is used with other traditional biomarkers [99,100]. Multiple studies and different methodological approaches have confirmed that exosomal HER2-positive exosomes reflect tumor progression in breast cancer [101]. This was confirmed by experimental data from Jia et al., who used Raman spectroscopy and exosome-based machine learning to predict the efficacy of breast cancer treatment and achieved a 0.89 AUC value [102]. Furthermore, another clinical study confirmed that exosomal CD44 enrichment promotes chemoresistance in doxorubicin-treated breast cancer cells [103]. Extending beyond cancer applications, hepatocyte-derived exosomal glucose transporter1 (GLUT1) can be used as a biomarker to identify the risk of non-alcoholic steatohepatitis (NASH) and diagnose stage two liver fibrosis [104]. Many clinical and preclinical studies have demonstrated that exosomal ACE2-containing defensomes function as decoys to prevent SARS-CoV-2 infection [105,106]. The clinical utility of exosomal caveolin-1 indicates biomarker potential in ovarian, bladder, and prostate cancers [107,108]. A 2021 study by Yang et al. identified that exosomal ALIX was a diagnostic biomarker for pancreatic cancer with a high AUC, particularly when combined with a traditional biomarker [109]. Taken together, these findings position the exosomal membrane as a comprehensive molecular diagnostic platform for disease-specific biomarkers (Table 1).

### 2.2. Exosomal Nucleic Acid Cargo in Disease Pathogenesis

Exosomes contain many nucleic acids that function as biomarkers for disease diagnosis, particularly focusing on four critical exosomal RNA types Table 2). Exosomal miRNAs, which regulate gene expression through post-transcriptional mechanisms, are the most extensively investigated category of regulatory exosomal RNAs [145]. Exosomal circRNAs function as miRNA sponges and demonstrate stability within exosomes due to their circular structure [146]. Exosomal tRNA-derived fragments (tRFs) have emerged as promising biomarkers, with studies showing that specific 5′-tRFs and 3′-tRFs are differentially expressed in various cancers [147]. Many studies have confirmed that exosomal snoRNAs and Y RNAs can be used as disease biomarkers across multiple pathological conditions [148,149]. The protective lipid bilayer structure of exosomes confers remarkable stability to their RNA cargo, preventing degradation by extracellular RNases and enabling reliable detection in body fluids. The clinical translation of these biomarkers is supported by their accessibility through liquid biopsies. Exosomal RNAs can be detected in plasma, serum, urine, and other body fluids. This offers non-invasive diagnostic approaches to cancer and other diseases. This combination of stability, specificity, and accessibility positions exosomal nucleic acids as next-generation biomarkers for precision medicine applications.

Exosomes, which consist of tumor-derived DNA, pathogen-specific RNA, and host-response signatures, collectively offer comprehensive understanding of disease states. For instance, exosomes derived from tumors contain double-stranded DNA that reflects the mutational status of progenitor tumor cells and represents the entire genome [150]. Specific diagnostic indicators are demonstrated by viral infections through the use of distinctive exosomal genomic signatures. This has been verified by numerous studies that have examined the Epstein–Barr virus (EBV), Hepatitis B virus [151], and human papillomavirus (HPV). Conversely, the RNA derived from bacterial, fungal, and parasitic sources in exosomes may be employed as a diagnostic tool for infectious diseases. This includes Plasmodium falciparum, mycobacterium tuberculosis [152], and Leishmania.

**Table 2:** Exosomal nucleic acid cargo in disease pathogenesis.

Functional Category	Disease-Relevant Exosomal Elements	Molecular Mechanisms	Disease Applications	Therapeutic/Diagnostic Potential
Regulatory RNA	miRNA [145], circRNA [146], tRFs [147], rRFs [153], snoRNA [148], Y RNA [149]	Functional Activity in Recipient Cells [145] mtDNA Released via Exosomes [154] Protected Transport [146] Targeted Delivery [147] Pathogen Transmission/Immune Evasion [151,152,155]	Cancer [145,146,147,150,156,157] Infectious Disease [151,152] Inflammatory Disease [149] Metabolic and Organ-Specific Diseases [154]	Immunomodulation [150] Therapeutic targets [157] Diagnosis of inflammatory and autoimmune diseases [149,154,158] Diagnosis of infectious diseases [151,152,155] Early detection and diagnosis [147,148,157]
Protein-Coding RNA	mRNAs [157]			
Pathogen-Derived RNA	Viral RNA [155], Bacterial RNA [152], Parasitic RNA [152]			
Genomic Material	Viral DNA [151], gDNA [150], mtDNA [154], ecDNA [159]			

### 2.3. Exosomal Protein Cargo in Disease Pathogenesis

Exosomal proteins can function as vital biomarkers for the diagnosis of diseases, as they reflect pathological states and cellular integrity. The protein cargo in exosomes can demonstrate how these vesicles facilitate disease spread through distinct mechanisms. Pathological protein propagation represents a key mechanism where exosomes are used as vectors for misfolded and aggregated proteins. These proteins include amyloid-β oligomers [160], TDP-43 [161], and LRRK2 [162], which are implicated in neurodegenerative diseases. Many studies demonstrate that exosomes from Alzheimer patients’ brains contain high levels of amyloid-β oligomers and can act as vectors to transfer toxic species [160]. Similarly, TDP-43 was identified in secreted exosomes from neurons, and the levels of full-length exosomal TDP-43 and C-terminal fragment species were upregulated in the brains of human patients with amyotrophic lateral sclerosis [161]. Exosomal β-catenin facilitates cellular signaling dysregulation [163]. Many studies have confirmed the presence of exosomal metabolic regulation proteins such as interleukin [164], TNF [165], FABP [166], PCSK9 [167], and adiponectin [168]. The ability of these exosomal proteins to activate inflammatory cascades is well confirmed. For example, exosomes can carry anti-inflammatory cytokines to inhibit the proliferation of T cells and B cells [169].

The potential for disease diagnosis and monitoring is substantial due to the diverse protein composition of exosomes. Exosomes have immune system modulation proteins, such as MPO [170], Nef [171], and NS1 [172]. Their presence provides insights into inflammatory states and immune dysfunction. A 2022 investigation by Han et al. showed that MPO is enriched in plasma-derived exosomes during the early phases of deep venous thrombosis (DVT), which promotes vascular endothelial cell injury and apoptosis [170]. Exosomal NS1 may serve as a diagnostic biomarker for dengue and Zika virus infections by enabling detection of the conserved NS1 glycoprotein. Separately, exosomal Nef may serve as a novel diagnostic indicator of HIV/SIV infection [171,172]. Cellular stress and structural alterations associated with disease progression are indicated by cellular structure and transport proteins, including annexin [173], Rab [174], fibronectin [175], EpCAM [100], and Glypican-1 [35]. Experimental data from Kulkarni et al. validated the existence of fibronectin in exosomes, which facilitates HIV-1 viral transfer from infected dendritic cells to T cells [175]. Furthermore, lysosomal function can be reflected in the hexosaminidase enzyme system of exosomes [176]. Exosomal protein profiling may eventually provide valuable diagnostic and prognostic information across multiple disease contexts. This potential is demonstrated by ongoing clinical trials to validate exosomal biomarkers to enhance diagnostic accuracy and predict treatment responses despite these limitations (Table 3).

### 2.4. Exosomal Lipids in Disease Pathogenesis

Exosomal lipids represent a critical component of the cellular structure that serves both structural and functional roles in disease diagnosis. The membrane structure and dynamics of exosomes are primarily governed by key lipid classes, including ceramides, sphingomyelins, cholesterol, glucosylceramides, and lactosylceramides. These lipids form the characteristic lipid bilayer that not only provides structural integrity but also influences membrane fluidity, protein integration, and vesicle stability in various biological fluids. Exosomal ceramides have dual functions as structural components and bioactive signaling molecules. Their levels exhibit considerable alterations due to disorders such as cancer, neurodegeneration, and metabolic diseases [23]. The exosomal sphingolipid metabolism pathway, encompassing sphingomyelin and its derivatives, becomes dysregulated in the face of numerous diseases. This dysregulation makes these lipid species valuable biomarkers for disease detection and monitoring [185]. Gangliosides, complex glycosphingolipids, are enriched in exosomes derived from neural tissues and serve as specific biomarkers for neurological diseases and brain tumor diagnostics [186].

The lipid composition of exosomes reflects the metabolic state of their parent cells and provides insights into disease-associated metabolic dysfunction. The cholesterol content within exosomal membranes influences membrane properties and protein function, with altered cholesterol levels observed in cardiovascular diseases, metabolic syndrome, and certain cancers [187]. The presence of specific glycolipids such as glucosylceramides and lactosylceramides in exosomes indicates active glycosphingolipid metabolism. This pathway is frequently disrupted by lysosomal storage disorders, diabetes, and inflammatory conditions [188,189]. These lipid biomarkers offer several advantages for disease diagnosis, including their stability within the protective exosomal membrane environment, their resistance to enzymatic degradation in biological fluids, and their reflection of cellular metabolic status. The diagnostic potential of exosomal lipids is further enhanced by their tissue-specific distribution patterns and disease stage-dependent alterations. This enables their use for early disease detection, prognosis assessment, and therapeutic monitoring. Advanced lipidomics approaches allow for comprehensive profiling of exosomal lipid signatures, providing personalized diagnostic information that complements protein and nucleic acid biomarkers for precision medicine applications (Table 4).

## 3. Clinically Exosomal Biomarkers

Exosomes have become diagnostic tools in clinical diagnostics, providing non-invasive access to disease-specific molecular cargo across a variety of disease categories (Table 5). Biomarkers in cancer, neurodegenerative diseases, and rare genetic disorders are validated by exosomes as demonstrated in sialidosis where, the deficiency of neuraminidase 1 leads to excessive lysosomal exocytosis and exosome release. A recent study using Neu1−/− models of fibroblasts found that exosomes enriched with TGF-β1, WNT3a, WNT5a/b, and β-catenin led to systemic fibrosis [198]. In Fabry disease, it was revealed that exosomes laden with profibrotic signals (TGF-β, WNT5a/b) or glycosphingolipids (Gb3) were linked to endothelial dysfunction [199,200]. Rare metabolic disorders, including Pompe disease, reveal exosomal miR-206 dysregulation, offering it as a non-invasive biomarker [201].

Exosomal phosphorylated Tau (T181) achieves high diagnostic accuracy for Alzheimer’s disease in neurodegenerative disorders [37]. Recent studies have demonstrated that exosomes can function as both disease mediators and biomarkers. For example, plasma exosomes in Parkinson’s disease contain α-synuclein oligomers and dysregulated miRNA [202]. Alzheimer’s disease is distinguished from controls by CSF and blood exosomes that are enriched with p-Tau181 and oAβ [203]. Consequently, exosomes exacerbate neurodegeneration through many mechanisms. This includes direct toxicity of mitochondria by α-synuclein and oAβ, as well as the stimulation of the inflammatory response and the spread of prion-like particles. In cancer, serum EGFRvIII constitutes active abnormal variants of the epidermal growth factor receptor (EGFR). Exosmal EGFRvIII is capable of detecting glioblastoma recurrence with high sensitivity in cancer through the use of exosomes [204].

In addition to neurodegeneration and oncology, exosomes provide insight into the pathogenesis and diagnostics of systemic diseases. Exosomal miR-122 and sphingolipids are associated with alcoholic liver fibrosis in liver pathologies [205], whereas miR-34a indicates early metabolic dysfunction in non-alcoholic fatty liver disease (NAFLD) [206]. Studies using animal models demonstrate that urinary exosomal TGF-β1 can predict diabetic nephropathy progression [207]. Separately, miR-29c is used as a diagnostic of fibrosis in chronic kidney disease [208]. Exosomal miR-223 is elevated in respiratory conditions such as asthma [209], while autoimmune disorders like autoimmune thyroid diseases exhibit exosome-mediated IL-1β/IL-6 activation [210]. In addition, the diagnostic potential of exosomal miR-155 in the assessment of hepatic fibrosis is superior to conventional biomarkers. Exosomal miR-155 demonstrated exceptional diagnostic performance in a clinical cohort that included 94 patients with liver fibrosis and 50 healthy controls [211]. In septic cardiomyopathy, low levels of exosomal miR-150-5p are linked to adverse outcomes, while overexpression of miR-150-5p mitigates apoptosis in sepsis-induced myocardial depression [201,212,213]. These developments emphasize the flexibility of exosomes in connecting molecular mechanisms to clinical diagnostics.

**Table 5:** Clinical exosomal biomarkers for different disorders with associated clinical trials.

Disease	Exosomal Biomarker(s)	Clinical Performance (Exosomal)	Standard Diagnostic	Advantages of Exosomal Biomarkers	Clinical Trials
Pancreatic Cancer	Glypican-1 (GPC1) [35,60]	Sensitivity: 0.88 Specificity: 0.86 AUC: 0.93	Serum CA19-9	Detects early-stage disease	NCT02393703, NCT06388967, NCT03791073,
Prostate Cancer	ExoDx Prostate IntelliScore (EPI) [65,66]	NPV: 91.3%	PSA and biopsy	Reduces biopsies by 27–30% and higher negative predictive value	NCT03235687, NCT02702856, NCT04556916
Alzheimer’s Disease	Tau and toxic amyloid-β oligomers [37,203]	96.4% correct classification	CSF biomarkers (p-Tau181, Aβ42, t-tau)	Allows detection years before clinical symptoms	NCT04388982
Breast Cancer	HER2 exosomes [101,102]	AUC: 0.89 (treatment response)	Tissue biopsy and IHC	Real-time monitoring; predicts therapy efficacy	NCT01840306, NCT01344109
Parkinson’s Disease	α-synuclein oligomers [214]	Sensitivity: 70.1% Specificity: 52.9% AUC: 0.654	Dopamine transporter (DAT) scan	detects pre-symptomatic stages	NCT05320250
Hepatic Disease	Exosomal miR-155 [211]	93.6% sensitivity and 94% specificity (AUC: 0.971)	CIV, Hyp, AST	Clinical indicators of different degrees of hepatic necrosis and fibrosis	NCT06342414, NCT05871463
Diabetic Nephropathy	Urinary exosomal WT-1 [207]	Predicts progression	Urinary albumin/creatinine ratio	Early prediction of renal injury	NCT06123871

## 4. Translational Technologies

The transition of exosome research from bench to bedside hinges on overcoming significant technological and methodological challenges. Exosomes show promise in kidney transplantation monitoring and Alzheimer’s disease diagnosis [215]. Moreover, they are very useful in the diagnosis of cardiovascular diseases and renal fibrosis [216,217]. Their capacity for multi-marker profiling further extends this utility to diagnose many diseases, including cancer [218]. While exosomes hold immense promise as biomarkers and therapeutic tools, their clinical implementation requires robust isolation techniques, sensitive analytical platforms, and standardized protocols. This section explores the current state of exosome-based technologies and the barriers to widespread adoption in medical laboratories.

### 4.1. Isolation and Standardization

**Figure 4:**
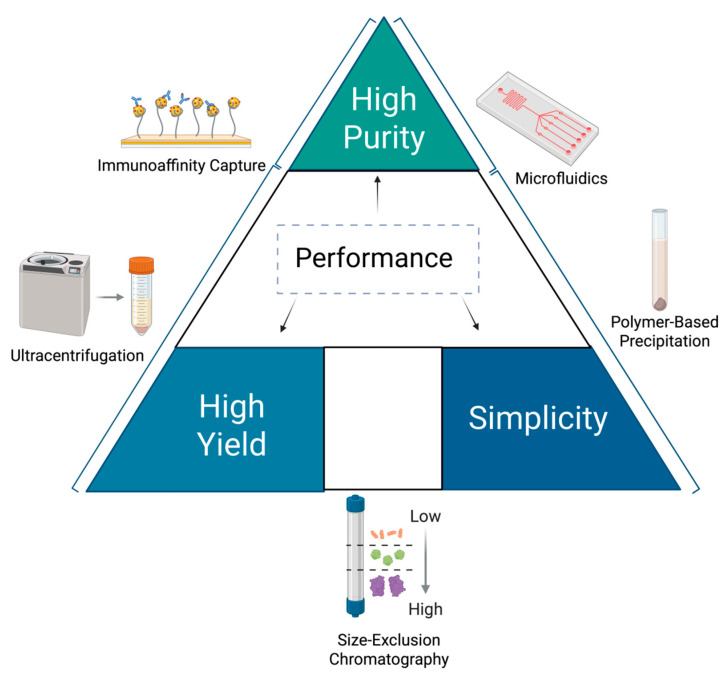
Method selection for exosome isolation based on purity, yield, and simplicity.

Isolating exosomes with high purity and yield remains a critical challenge in clinical applications (Figure 4) [219]. Ultracentrifugation, the traditional gold standard, offers high purity but is time-consuming and may compromise exosome integrity due to mechanical shear forces [220]. Alternative methods, such as size-exclusion chromatography, polymer-based precipitation, and immunoaffinity capture, provide faster processing but vary in specificity and scalability [221]. Other alternative methodologies pose special difficulties. As an illustration, size-exclusion chromatography is limited by a low yield for small-volume samples, which is an important consideration in diseases. Moreover, in polymer-based precipitation, the co-precipitation of contaminants in the size range of exosomes is the challenge. Furthermore, in immunoaffinity capture, variation in exosome size and epitope variability is one of the challenges. Despite this, the technique remains highly effective for isolating exosomes based on known surface markers of exosomes. Current isolation methods encounter substantial obstacles in distinguishing exosomes from apoptotic bodies and ectosomes because of their overlapping sizes, densities, and sedimentation characteristics [222]. Currently, emerging microfluidic technologies enable high-throughput isolation with minimal sample volumes, making them attractive for diagnostic workflows [223]. The lack of standardized protocols across laboratories introduces variability in exosome recovery and characterization. To address these issues, the International Society for Extracellular Vesicles, initiatives like the EV-TRACK consortium, and the MISEV and ISO/TC276 guidelines aim to establish best practices for exosome research [224,225].

### 4.2. Analytical Platforms

**Figure 5:**
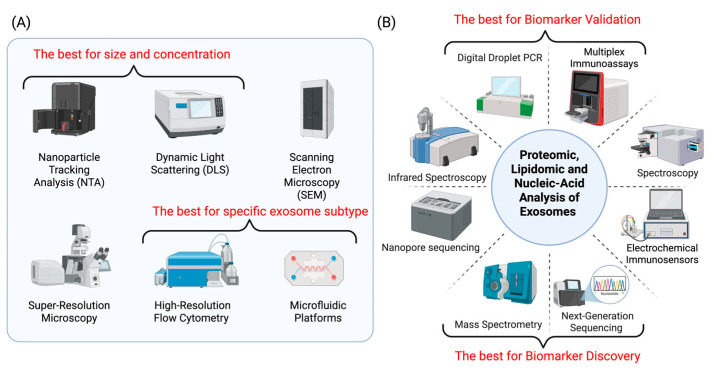
Comprehensive analytical methods available for physical characterization (**A**) and molecular analysis (**B**) of exosomes.

The diagnostic efficacy of exosomal biomarkers in clinical laboratories is dependent on their sensitive and multiplexed detection. The heterogeneity of exosomes is a critical characteristic that is indicative of their diverse molecular compositions and origins. This heterogeneity is being increasingly revealed through advanced characterization techniques. These include atomic force microscopy (AFM) and high-resolution flow cytometry platforms, including hapFCM and NanoFCM, which facilitate the analysis of individual exosomes [226,227]. At the single particle level, interferometric nanoparticle tracking analysis (iNTA) offers high-precision dimension and refractive index measurements of nanometer-sized particles, such as exosomes [228]. Dynamic Light Scattering (DLS) has limitations toward exosomes, including intensity-weighted bias and limited resolution for complex mixes. Nevertheless, its speed, sensitivity, and minimal sample requirements make it appropriate for routine exosome analysis [229]. Exosome ultrastructure can be visualized, and dynamic processes can be tracked using super-resolution microscopy technologies such as SMLM, DNA-PAINT, and STED. Microfluidic technologies such as immunoaffinity, electrical, physical, and magnetic approaches represent a transformative approach to exosome characterization (Figure 5A).

In exosomal proteomics, advanced immunoassays and mass spectrometry (LC-MS/MS) support multiplexed and high-throughput protein analysis. By employing data-independent acquisition workflows, it is possible to quantify thousands of exosomal proteins across multiple samples without the need for antibodies. Additionally, advanced immunoassay platforms, including microfluidic chips, chemiluminescence assays, and bead-based multiplex arrays, enable the targeted analysis of low-abundance markers in clinically relevant volumes. The detection limits for exosomal markers have been reduced to as low as 0.7 × 10^4^ particles/mL by incorporating surface-enhanced Raman scattering (SERS) and using antibody-modified nanoparticles [230,231]. In exosomal nucleic acid detection, digital droplet PCR (ddPCR), next-generation sequencing (NGS), and Nanopore Sequencing have substantially improved the detection of exosomal nucleic acids by providing superior specificity, sensitivity, and analytical capabilities compared to conventional techniques. In exosomal lipidomic and metabolomic analysis, MALDI-TOF and Raman spectroscopy are employed, which offer potent molecular insights into exosomes [197]. Additionally, the approach is shifted toward vibrational spectroscopic analysis by incorporating FTIR and Raman spectroscopy into exosome research. This synergistic approach enhances molecular characterization by leveraging the complementary qualities of both approaches [232] (Figure 5B).

CRISPR/Cas systems can be designed to target nucleic acids or proteins that are specific to exosomes, thereby enabling the highly specific capture and identification of exosomes derived from tumors. To illustrate, CRISPR-Cas12a and Cas13a have been integrated with aptamers and hybridization chain reaction (HCR) to develop ultrasensitive assays. These assays have achieved detection limits as low as 10^2^ particles/µL, surpassing conventional methods such as ELISA [233,234]. In light of recent advancements in exosome research, researchers may develop a more sophisticated comprehension of disease mechanisms and identify highly specific biomarkers. This is achieved by integrating data from genomics, proteomics, transcriptomics, and metabolomics and utilizing AI for data interpretation and integration. The development of personalized therapeutic and diagnostic strategies is further powered by AI, thereby positioning exosome analysis at the vanguard of precision medicine [235].

## 5. Technical and Implementation Challenges

**Figure 6:**
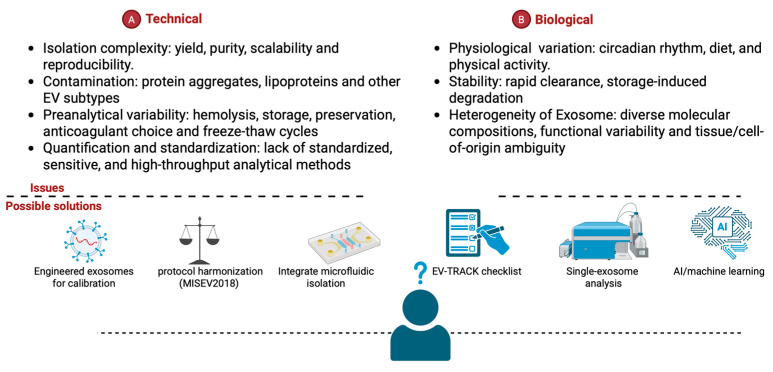
Key technical (**A**) and biological (**B**) challenges encountered in exosome studies, along with corresponding “possible solutions” depicted below each panel.

Translating the mechanistic understanding of exosome biomarkers into clinical laboratory practice is a complex process fraught with real-world obstacles. While exosome-based diagnostics hold significant promise, their clinical implementation is currently limited by several critical factors (Figure 6A,B). Notably, only one has received FDA’s breakthrough device designation for exosome-related assays—ExoDx Prostate IntelliScore. This test uses urinary exosomal RNA for prostate cancer risk stratification, and has demonstrated clear clinical utility, notably reducing the need for invasive biopsies [236]. Despite this advance, few clinical laboratories offer exosome-based tests, reflecting the field’s nascent state and the many barriers to adoption. Regulatory agencies such as the FDA and EMA should develop frameworks for exosome-based diagnostics and therapeutics, emphasizing the need for clear definitions of exosome composition and potency [237]. Ethical considerations, including patient consent for the use of exosome-derived data, also require attention. Additionally, the high costs associated with exosome isolation and analysis may limit accessibility, particularly in resource-limited settings. Addressing these multifaceted challenges will require interdisciplinary collaboration, investment in scalable technologies, and robust evidence demonstrating the cost-effectiveness of exosome-based diagnostics compared to conventional methods.

Several implementation challenges account for this limited uptake. Without standardized operating procedures and robust validation studies, it is difficult to ensure consistency and reliability across different laboratories and platforms. Beyond these practical hurdles, the integration of exosome technologies into routine clinical practice is further complicated by biological and technical challenges. Moreover, accessibility may be restricted by the high costs related to exosome isolation, analysis, and the development of specific tests. Accessibility may be restricted by the high costs related to exosome isolation and analysis, as well as the development of specific tests. On the technical side, isolation procedures are often complex, affecting yield, purity, and scalability. A major complication is contamination from protein aggregates and lipoproteins. Efforts are focused on the development of engineered exosomes for calibration and single-exosome analysis technologies [25,238]. Moreover, efforts are directed toward AI-driven machine learning approaches to enhance diagnostic accuracy and clinical utility [239]. Multicenter studies with standardized protocols are essential to establish clinically relevant cutoff values and validate the broader adoption of exosome biomarkers. Ultimately, overcoming these obstacles will be crucial to realizing the full potential of exosome-based diagnostics as next-generation tools in clinical laboratories.

### 5.1. Preanalytical Variables of Exosomes

The procedure for sample collection, processing, storage, and handling may have an effect on exosome yield, integrity, and biomarker stability, which could compromise the detection of disease biomarkers. A key investigation determined the occurrence of high levels of variability in exosome-based assays in the preanalytical phase [240]. There are many exosomal markers that are highly sensitive to preanalytical conditions, especially the storage temperature, including exosomal markers CD63 and CD9. The variability and low repeatability rate in exosome cargo composition can be greatly influenced by biofluids, collection tubes, and timing of sample processing [241]. Data from a recent study revealed that the concentration of large EVs in plasma was nearly two-fold higher in heparinized plasma than in other anticoagulants [242]. Further contributing to inter- and intra-individual heterogeneity are physiological elements, including circadian rhythm, diet, and physical activity. A recent study confirmed that exosome number and size distribution may vary [243], and that physical activity can momentarily raise circulating exosome levels. Exosome stability is substantially influenced by storage temperature and length; 4 °C storage results in lower particle counts and changed surface markers, whereas long-term preservation is best served at −80 °C [244]. Evidence from multiple studies suggests that repeated freeze–thaw cycles may lead to exosome degradation and integrity loss. In a recent study comparing fresh exosomes with frozen ones, it was found that the diameter of exosomes increased by 10% at 4 °C and 25% at −80 °C [245].

The use of cryoprotectants (e.g., DMSO) and storage in native biofluids may cause cytotoxicity in the cells and lead to exosomal destabilization and disruption [245]. Based on many studies, preanalytical processing of samples affects exosome yield, purity, and miRNA content. These effects are due to different filtration, centrifugation, and protease or RNase inhibitor addition protocols [246]. Under these conditions, best practice recommendations call for processing samples as soon as they are obtained to minimize freeze–thaw cycles and precisely record all preanalytical variables in experimental reports. To guarantee repeatability and dependability in both clinical and research environments, two initiatives are needed. These are harmonization of preanalytical procedures and the evolution of strong quality control mechanisms, both of which are desperately needed.

### 5.2. Clinical Laboratory Standardization for Exosomes in Diagnostics

The clinical translation of exosome-based diagnostics and therapeutics is fundamentally limited by the lack of standardized protocols for exosome isolation, characterization, and reporting. Despite the proliferation of isolation techniques—including ultracentrifugation, size-exclusion chromatography, polymer-based precipitation, immunoaffinity capture, and microfluidics—there is no universally accepted “gold standard” method. Each approach presents trade-offs between yield, purity, scalability, and preservation of exosomal integrity. To address this heterogeneity, the International Society for Extracellular Vesicles (ISEV) released and regularly updates the Minimal Information for Studies of Extracellular Vesicles (MISEV) guidelines, most recently in 2018 and 2023 [224,247]. These guidelines advocate for transparent reporting of isolation and characterization methods, recommend the use of both positive markers including tetraspanins and negative markers such as albumin in order to assess sample purity, and encourage the use of functional assays to confirm EV-specific activity. The EV-TRACK knowledge base and ISO/TC276 standards further support protocol harmonization and data transparency. Additionally, the development of synthetic exosomes, including NIST-traceable vesicles spiked with miR-21 calibrators, has significantly improved assay precision, reducing the inter-laboratory coefficient of variation (CV) [248]. To guarantee consistent performance across healthcare settings, external quality assessment (EQA) programs, including CAP proficiency surveys, are implemented for clinical purposes. Despite these advances, inter-laboratory variability remains a major barrier, as even minor deviations in isolation or handling can significantly alter exosome yield, composition, and biomarker readouts. This challenge is compounded by the fact that the guidelines and standards (such as MISEV) refer broadly to EVs. Nevertheless, numerous studies—including those that adhere to the MISEV guidelines—use the term “EVs” to refer to exosomes, microvesicles, and other vesicle types as a result of technical constraints in isolation and characterization.

## 6. Summary and Future Directions

Exosomes are a subset of extracellular vesicles (EVs) ranging from 30 to 150 nm and have been recognized as cellular debris, mediators of intercellular communication, and promising clinical biomarkers [1]. Exosomes have a unique biogenesis that enables selective cargo loading of proteins, lipids, and nucleic acids, depending on signaling needs [2,9]. The composition of exosomes is actively influenced by selective cargo loading mechanisms that respond to the cell’s physiological or pathological state, enabling these exosomes to function as precise carriers of molecular information. Therefore, they are capable of reprogramming recipient cells and influencing disease processes at different levels. This significant transformation was triggered by recent developments in analytical technologies including single-exosome analysis and integrated platforms [226,227]. Therefore, exosomes have emerged as candidate targets for non-invasive diagnostics, where they may offer superior sensitivity and specificity compared to traditional biomarkers [31,32,33,34,35,37,38,41]. Additionally, exosomes are present in many body fluids and hold the ability to cross biological barriers, making them an optimal choice for the diagnosis of many diseases [17,18,19].

The molecular diagnostics field has been improved by exosome biology, which identifies them as dynamic carriers of disease-specific proteins, nucleic acids, and lipids. Exosomes are very diverse in terms of diagnosis, ranging from oncogenic signaling cascades to neurodegenerative protein aggregation (Table 1, Table 2, Table 3 and Table 4). In the future, exosomes have the potential to transform liquid biopsies; however, this transition is impeded by numerous obstacles, such as heterogeneity, preanalytical variability, and the absence of standardized isolation protocols. To ensure that exosome-based diagnosis become accepted practices in medical laboratories, future efforts should focus on international validation studies, automation, and cost-effective solutions. Advances in exosome analysis now leverage artificial intelligence (AI) for improved clinical predictions, with neural networks evaluating many exosomal features to accurately forecast cancer [249,250]. Additionally, the development of synthetic exosomes, including NIST-traceable vesicles spiked with miR-21 calibrators, has significantly improved assay precision, reducing the inter-laboratory coefficient of variation (CV) [248]. These advancements emphasize the significance of computational biology and standardized reference materials in improving the clinical utility and reproducibility of exosome-based diagnostics.

Beyond diagnostics, exosomes are emerging as powerful therapeutic vehicles due to their natural biocompatibility and targeting capabilities [251]. Their inherent ability to evade immune clearance, which is mediated by surface markers like CD47, makes them ideal for drug delivery [88]. Researchers are engineering exosomes to carry siRNA, chemotherapeutics, and gene editing tools, with several candidates already in preclinical and clinical trials [252]. For example, exosome-based therapies for lysosomal storage diseases such as Gaucher disease are showing promise in early-stage trials [253]. However, challenges remain in large-scale manufacturing, ensuring batch-to-batch consistency, and meeting regulatory requirements. The therapeutic potential of exosomes also extends to regenerative medicine, where they may serve as acellular alternatives to stem cell therapy. As the field progresses, collaborations between biotech companies and regulatory agencies will be crucial to translating these innovations into approved treatments.

Exosome research is currently at an important turning point in bridging the gap between discovery and clinical application by using different advanced translational technologies [197,226,227,228,229,230,231]. Significant progress has been made in these translational technologies. As biomarkers for the diagnosis of Alzheimer’s, cardiovascular diseases, renal fibrosis, cancer, and others, as well as for the monitoring many diseases, exosomes possess immense translational potential [37,198,199,200,201,202,203,204,205,206,207,208,209,210,212,213,254,255,256]. Their implementation is limited by their isolation and analytical processes. However, many advanced platforms may offer multiplexed detection, including high-resolution flow cytometry, super-resolution microscopy, SERS, ddPCR, NGS, and CRISPR/Cas systems [197,226,227,228,230,231,233,234,235,238,257,258]. The isolation, characterization, and reporting of exosomes requires a comprehensive regulatory pathway and a universally practicable protocol. Currently many organizations are attempting to establish widely accepted protocols, such as ISO/TC276, EV-TRACK, and the MISEV guidelines [224,225]. In order to maximize the potential of exosomes in diagnosis, it is necessary to address clinical validation, standardization of isolation and analysis techniques, and regulatory harmonization.

## 7. Conclusions

The future is going to be significantly impacted by the expansion of exosome research and clinical use. A fundamental change from a discovery-centric to an integration-centric paradigm is necessary to accomplish this, as this review has argued. Exosome-based diagnostics will be made even more sensitive, scalable, and user-friendly. This will be driven by the implementation of next-generation separation and analytical technologies, including microfluidic platforms, AI-driven multi-omics integration, and CRISPR-based biosensors [235]. Exosomes have garnered significant attention in recent years due to their potential for therapeutic applications and diagnostic applications. Because of their biocompatibility, minimal immunogenicity, and ability to traverse biological barriers, exosomes are more effective than whole-cell treatments for drug delivery. Since exosomes are produced by endogenous cells, they will not trigger an immune response, in contrast to many synthetic carriers that may contain cationic lipids or polymers, which can lead to cellular stress and membrane rupture. This is due to the fact that they are able to naturally target specific cells and surface proteins and ligands facilitate their identification of and connection with these cells. Currently, there are numerous clinical trials underway for this purpose.

## Figures and Tables

**Table 1:** Exosomal membrane proteins in disease pathogenesis.

Functional Category	Disease-Relevant Exosomal Elements	Molecular Mechanisms	Disease Applications	Therapeutic/Diagnostic Potential
Cell Adhesion and Migration	Tetraspanins [110,111,112], Integrins [84,113,114], ICAM-1 [93,94,95], EpCAM [99,100], L1CAM [115,116], Cadherin [117], CD44 [103], CLDN4 [118], Glypican-1 [35,60], CEACAM5/6 [119]	Metastasis by mediating uptake [114,120] Systemic immunosuppression [86] Immune evasion [87] Induces regulatory cytokines [96] Inhibiting viral entry [105]	Organotropism in many cancers [114] Tetraspanin and metastasis [120] Distinguish benign from cancerous [60] Immunosuppressive correlation [86] Resistance to therapies [105] Response to drug assessment [86]	Exosome inhibitors may inhibit metastasis [87,105,114] Engineered ACE2-exosomes for COVID-19 [106]
Immune Modulation and Antigen Presentation	MHC-I/II [89,90], CD86 [121,122], TIM-3 [92], CD80 [123], PD-L1 [85,86], CD47 [87,88], Galectin-9 [96], CD40/CD40L [97], FASL and TRAIL [98], Alpha-Synuclein [124], CTLA-4 [91,125], CD59 [126]			
Signaling Receptors and Disease-Associated Proteins	HER2 [101,102], PDGFRβ [127], EPHA2 [128], PSGR [129], MET [130], MUC1 [131], Prominin-1 [132], Notch receptors [133], LRP1 [134], GPNMB [135], ACE2 [105,106], GLUT-1 [104], ApoE [136], CD74 [137], CXCR [138], TLR [139]			
Vesicle Biogenesis, Structure, and Trafficking	Flotillin-1/2 [57], Annexins [140,141], Caveolin-1 [107,108], ALIX [109], HRS [142], Tetherin [143], LAMP1 [144]			

**Table 3:** Exosomal protein cargo in disease pathogenesis.

Functional Category	Disease-Relevant Exosomal Elements	Molecular Mechanisms	Disease Applications	Therapeutic/Diagnostic Potential
Pathological Protein Propagation	Tau [177], Amyloid-β [160] TDP-43, [161], LRRK2 [162]	Spread of Pathogenic Proteins [160,177] Delivery of Signaling and enzymes [164,168,176,178] Direct Ligand–Receptor Signaling [167,178] Antagonism of Signaling Molecules [163] Pathological Protein Clearance [161]	Neurodegenerative Diseases [161,162,177] Cancer [161,179,180] Inflammatory and Autoimmune Diseases [165,181] Infectious Diseases [172,175] Metabolic Diseases [166,168] Cardiovascular Diseases [167,170] Immune Regulation [161,169]	Targeting Pathogenic Exosomes [177] Engineering Exosomes as Therapeutics [176] Cancer Early Detection [35] Cancer Chemotherapy Response [173]
Oncogenic Signaling and Metabolic Regulation	β-catenin [163], TGF-β [179], LDH-C4 [182], PKM2 [183], EGFR Hexosaminidase A [176], FABP4 [166], PCSK9 [167], Adiponectin [168]			
Immune System Modulation	IL [164], TNF [178], Calprotectin [165], Nef [171], NS1 [172], MPO [170],			
Cellular Structure and Transport	Annexin [173], Rab [174], Fibronectin [175], EpCAM [100], Glypican-1 [35], CA-125 [184]			

**Table 4:** Exosomal lipids in disease pathogenesis.

Functional Category	Disease-Relevant Exosomal Elements	Molecular Mechanisms	Disease Applications	Therapeutic/Diagnostic Potential
Membrane Structure and Dynamics	Ceramide [190], Sphingomyelin [191], Cholesterol [187], Glucosylceramide [189], Lactosylceramide [188], Gangliosides [186]	Eicosanoid Delivery Plasmalogen [192] Enrichment [193] Ceramide-Mediated Biogenesis/Signaling [191] Glucosylceramide Shuttling [189] Cholesterol Delivery [187] Malignancy Transfer [186]	Cancer [188,190,194,195] Multiple Sclerosis [191] Alzheimer’s Disease [187]	Inhibiting Exosome Biogenesis [190,191] Engineering Exosomes [189,193] Lipidomics-Based Liquid Biopsies [188,195]
Mitochondrial Function and Energy Metabolism	Cardiolipin [194], Fatty Acids [195]			
Cellular Signaling and Inflammation	Phosphatidylserine [196], Phosphatidic Acid [195,197], Eicosanoids [192]			
Metabolic Regulation	Plasmalogens [193]			

## Abbreviations

The following abbreviations are used in this manuscript: ACE2—Angiotensin-Converting Enzyme 2; ALIX—ALG-2 Interacting Protein X; AUC—Area Under the Curve; CEACAM—Carcinoembryonic Antigen-Related Cell Adhesion Molecule; CLDN4—Claudin-4; CTLA-4—Cytotoxic T-Lymphocyte-Associated Protein 4; CXCR—C-X-C Chemokine Receptor; DLS—Dynamic Light Scattering; EPCAM—Epithelial Cell Adhesion Molecule; ESCRT—Endosomal Sorting Complexes Required for Transport; FABP—Fatty Acid-Binding Protein; FTIR—Fourier-Transform Infrared Spectroscopy; GPNMB—Glycoprotein Non-Metastatic Melanoma Protein B; HER2—Human Epidermal Growth Factor Receptor 2; HRS—Hepatocyte Growth Factor-Regulated Tyrosine Kinase Substrate; ICAM-1—Intercellular Adhesion Molecule 1; LAMP1—Lysosomal-Associated Membrane Protein 1; L1CAM—L1 Cell Adhesion Molecule; LRP1—Low-Density Lipoprotein Receptor-Related Protein 1; MALDI-TOF—Matrix-Assisted Laser Desorption/Ionization Time-of-Flight; MPO—Myeloperoxidase; MUC1—Mucin 1; Nef—Negative Factor; NS1—Non-Structural Protein 1; NT-proBNP—N-Terminal Pro-B-Type Natriuretic Peptide; PD-1—Programmed Cell Death Protein 1; PDGFRβ—Platelet-Derived Growth Factor Receptor Beta; PKM2—Pyruvate Kinase M2; PSGR—Prostate-Specific G-Protein Coupled Receptor; Rab—Ras-Associated Binding Protein; STED—Stimulated Emission Depletion Microscopy; TGF-β—Transforming Growth Factor Beta; TIM-3—T-Cell Immunoglobulin and Mucin-Domain Containing-3; TNF—Tumor Necrosis Factor; VALID—Verifying Accurate Leading-Edge IVCT Development; Y RNA—Y-Chromosome RNA.
